# Revisiting the Role of Neighbourhood Change in Social Exclusion and Inclusion of Older People

**DOI:** 10.1155/2012/148287

**Published:** 2011-10-13

**Authors:** Victoria F. Burns, Jean-Pierre Lavoie, Damaris Rose

**Affiliations:** ^1^Centre de Recherche et d'Expertise en Gérontologie Sociale (CREGÉS), C.S.S.S. Cavendish, 5800 Cavendish Boulevard, Montréal, QC, Canada H4W 2T5; ^2^School of Social Work, McGill University, Montréal, QC, Canada H3A 2A7; ^3^École de Travail social, Université du Québec à Montréal, Montréal, QC, Canada H2L 4Y2; ^4^Centre Urbanisation Culture Société, Université INRS, 385 Rue Sherbrooke Est, Montréal, QC, Canada H2X 1E3

## Abstract

*Objective*. To explore how older people who are “aging in place” are affected when the urban neighbourhoods in which they are aging are themselves undergoing socioeconomic and demographic change. *Methods*. A qualitative case study was conducted in two contrasting neighbourhoods in Montréal (Québec, Canada), the analysis drawing on concepts of social exclusion and attachment. *Results*. Participants express variable levels of attachment to neighbourhood. Gentrification triggered processes of social exclusion among older adults: loss of social spaces dedicated to older people led to social disconnectedness, invisibility, and loss of political influence on neighbourhood planning. Conversely, certain changes in a disadvantaged neighbourhood fostered their social inclusion. *Conclusion*. This study thus highlights the importance of examining the impacts of neighbourhood change when exploring the dynamics of aging in place and when considering interventions to maintain quality of life of those concerned.

## 1. Introduction

A number of researchers have suggested that with advancing age, a person's geographical area tends to become increasingly limited in space [1–6]. Research that has explored the question of the meaning of place in different groups indicates that proximity of neighbours has a greater importance in the lives of older residents [7]. The neighbourhood is more significant for older people and the disadvantaged than for the younger and more affluent, who tend to develop social networks more diffuse in space [8, 9]. (Following current conventions, we use the terms “older person” and “older adult” in reference to people aged 65 years and over.) Moreover, the neighbourhood and the “home” become key elements in social life—social relations gradually become limited to people who live nearby—and also in defining one's sense of self, because the neighbourhood provides a number of identity markers [10]. Older people tend to be more reliant on their immediate environment as they are less likely to be involved in employment and have greater chance of becoming physically dependent [11]. Despite the growing body of aging-in-place research, social gerontology, hampered by static “environmental fit” models [12] has paid relatively little attention to the changes taking place in the neighbourhoods within which older people are aging and to how they experience these changes (submit to? actively participate in?…). It has been largely up to researchers in the geographies of aging to demonstrate the importance of neighbourhood change. Moreover, the social gerontology literature on aging in place and on the role of place in aging in old urban neighbourhoods—including a notable recent UK-Canadian comparison [13]—still focuses predominantly on neighbourhoods experiencing observable physical decline or mounting criminality. The latter could have negative impacts on older people's comfort level in their homes or on their ability to appropriate and navigate local public spaces [14, 15]. This focus is not surprising given that living in neighbourhoods of “multiple deprivation” can potentially reinforce the social exclusion of older people. However, it is also important to uncover possible dynamics of social exclusion of older people who find themselves living amidst growing affluence where they experience forms of place reshaping largely beyond their control, as in the case of neighbourhoods undergoing gentrification [16].

Gentrification is simultaneously a physical, economic, social, and cultural phenomenon classically defined in the literature as involving the “invasion” of previously working-class neighbourhoods by middle or upper-income groups and the subsequent displacement of many of the original residents [17]. (The use of the term “invasion” intentionally evokes the notion of invasion and succession developed by the Chicago School of urban sociology.) Debates and empirical research surrounding this topic now amount to a vast body of scholarship in urban geography and cognate fields; indeed, gentrification is often seen as the most important type of urban change across the global North over the past three decades [18]. This process involves a change in population characteristics with the arrival of younger, better educated people with higher incomes, a significant increase in the cost of housing (including house values, rents, property taxes), particular styles of commercial revitalization, increased traffic on neighbourhood commercial streets, and finally, displacement of former residents to more affordable neighbourhoods. 

Over the past 15 years or so, the forms taken by gentrification have diversified [19]. While private-sector actors (e.g., home renovators, landlords) still cause displacement, core city municipalities of large metropolitan areas are increasingly courting, even orchestrating gentrification. For example, they may facilitate new housing construction and the rebranding of neighbourhood commercial arteries [20, 21] so as to relaunch local economies, resolve fiscal crunches, and attract young and urbane singles and/or families, such that in some cases the long-standing trend for demographic aging of the innercity has been dramatically reversed. Consequently, scholarly debates are increasingly seeking to conceptualize and shed light on the various forms of “indirect” displacement that may be created when an existing population is not literally forced out of an area—because they live in social housing or are otherwise protected from displacement in the literal sense—but their local cultures and narratives of place, their access to familiar services, or their channels of local political representation are disrupted by the influx of younger, more educated and wealthier newcomers [22–25]. A complementary insight from the social determinants of health literature [26] is that discrepancies between personal income (low) and neighbourhood status (high) can be associated with poor health, especially for older people. These trends create a need to deepen our understandings of how gentrification can affect older people. 

Empirical work on its indirect negative effects on older people is as yet very sparse, but with a few insightful exceptions, such as that of Lehman-Frisch [27] alluding to commercial gentrification's culturally and economically exclusionary impact on long-term older adult residents of San Francisco and findings from Toulouse, France showing how neighbourhood revitalization may generate among older adults a sense of being out of place to the point that they are reluctant to venture out of their house [3]. In contrast, little attention has been paid to the potentially positive experiences of some older people in contexts of gentrification [28]. For instance, with gentrification comes an increase in real estate assets and it may give a greater sense of security due to increased numbers of people on local shopping streets, improved public facilities and services, and more opportunities to meet people. For these reasons, we decided to launch a study to explore older adults' perceptions of gentrification and to determine its effects on both their social exclusion and inclusion. Drawing on concepts of social exclusion, direct and indirect displacement, and attachment, this paper addresses how older people experience change in two contrasting neighbourhoods in Montréal, Canada: (1) La Petite-Patrie, a rapidly gentrifying neighbourhood and (2) Lower Notre-Dame-de-Grâce (NDG), a disadvantaged neighbourhood. This study forms part of a larger qualitative research project aiming to better understand the ways in which gentrification can contribute to the dynamics of social exclusion and inclusion of older people. As well as the Montréal study areas, the research has an international comparative dimension embracing two neighbourhoods in Toulouse, France (Minimes and Marengo). (A comparison including the two French neighbourhoods is beyond the scope of this paper.) The aims of this study were to answer the following questions.

1. What place does the neighbourhood have in the everyday lives of older residents? (What places do they frequent? Where are their social networks situated? What neighbourhood resources and services do they use?)
2. What neighbourhood changes do older residents notice?
3. How do neighbourhood changes affect older residents' experiences of social exclusion/inclusion?

## 2. Theoretical Framework

### 2.1. Social Exclusion

This research project is framed primarily by a conceptualization of the dynamics of social exclusion [29]. Social exclusion originated as a sociological concept, emerging from European policy circles, especially in the 1990s [30, 31] extending into gerontological research and public policy debates, especially within the context of the United Kingdom [14, 15, 32–34]. More recently, Billette and Lavoie [29] define social exclusion as a process of nonacknowledgement and deprivation of rights and resources of certain segments of the population (in this case, older adults) that takes the shape of power dynamics between groups with divergent visions and interests. Such processes result in inequities and lead eventually to isolation from society in seven dimensions: (1) symbolic exclusion (negative images, overrepresentations, and invisibility); (2) identity exclusion (multiple identities are dismissed and a person's identity is reduced to belonging to one singular group, for example, “old”, “frail”, “burdensome”); (3) sociopolitical exclusion (barriers to civic/political participation); (4) institutional exclusion (reduced access to services); (5) economic exclusion (lack of financial resources); (6) exclusion of significant social ties (absence/loss of social network); (7) territorial exclusion (reduced geographic living area, unsafe neighbourhood). Billette and Lavoie's definition puts forth two essential characteristics of social exclusion. First, it is a dynamic, fluid process rather than a static state. Second, since it is a multidimensional concept, it allows for a rich understanding of how social exclusion can be experienced across many facets of a person or population's life. For instance, in relation to gentrification, increases in rents and the changing commercial landscape may put financial strain on older people, especially those with modest incomes (economic exclusion). Older adults could also lose their political influence in relation to social planning (sociopolitical exclusion). The arrival of younger, more educated populations can reinforce certain stereotypes of older people, such as “slow”; “nosy”; “busy-bodies” of the neighbourhood (symbolic exclusion). Leaving a familiar neighbourhood or having friends and neighbours move away can also lead to social network exclusion. The dimension of territorial exclusion is of particular interest because neighbourhood change involving gentrification could lead to feelings of insecurity as familiar institutions disappear and the public spaces of everyday life take on a new look and “feel.” Territorial exclusion also shares dimensions with the useful geographic concepts of direct displacement (physically forced out of one's neighbourhood) and indirect displacement, as described above.

### 2.2. Attachment to Place

The concept of attachment is central to understanding how urban change can affect older adults. An individual's level of attachment to their environment will have a direct impact on how changes are experienced and perceived. This is especially the case for older people because, as mentioned above, the immediate environment becomes more important with age [8, 9]. Older people develop a sense of self-attachment, personal identity, and social differentiation through the relationship they construct and maintain with daily, “ordinary” spaces [35]. Therefore, understanding older people's attachment to place becomes a crucial element to understanding how they experience neighbourhood change.

It is important to make the distinction between place and space. Space refers to the physical location, whereas place can be thought of as a process and includes an integration of physical, social, emotional and symbolic aspects, interacting in different degrees [6, 36]. Several authors have since written on attachment to place [37–41] dating back to the path-breaking work of Rowles [42, 43] who developed a theory of *insideness* to conceptualize attachment to place, using three components: (1) autobiographical; (2) physical; (3) social. For Rowles, physical insideness is associated with living somewhere for long periods of time—the resident establishes a sense of environmental control or mastery by creating an idiosyncratic rhythm and routine. Social insideness evolves not only from everyday social exchanges and relationships but also from a sense of being well known and knowing others. Third, autobiographical insideness has been suggested to be the most relevant to describe older people's attachment to place because it is embedded in memories. As we age, these memories are recalled selectively in the creation of one's identity. Older people with strong ties to place are also reported to feel more in control, more secure and to have a positive sense of self. Attachment to place has also been studied by Rubinstein and Parmelee [40] and more recently by Sugihara and Evans [44] who make the link between older people's attachment to their dwelling, maintaining a positive self-image and maintaining their independence. Overall, in the past 30 years the study of attachment to place has captured the attention of scholars from geographical, gerontological, and environmental psychology perspectives [6, 37, 39, 45, 46], yet to date little has been written on what occurs when older people, who are aging in place, experience a neighbourhood that is itself undergoing change [14].

## 3. Methods

### 3.1. Study Design and Sample

We situated our research in an explorative, qualitative case study design [47], cases being the changing neighbourhood, the unit of analysis being the older person's personal experience of neighbourhood change. Case study methodology is suitable for studying complex and multifaceted social phenomena embedded in specific contexts [48]. Our overarching research question is concerned with how older adults experience different types of neighbourhood change, especially those involving gentrification. Exploring this complex issue necessitates recourse to multiple sources of evidence (e.g., document analysis, interviews with older people, and key informants), which is typical of case study methodology in the social sciences. For the Montréal component of the research, we selected two inner-city neighbourhoods (see descriptions below) where local community stakeholders were concerned about current or impending gentrification and how it could affect older people. Following a complete ethics review process by two university ethics boards (covering informed consent, confidentiality, respect, risks, and benefits, etc.), we conducted 30 semistructured face-to-face interviews with autonomous and mobile older adults aged from 68 to 95 years. All of our participants had lived in one of the two Montréal neighbourhoods for at least 10 years or did live previously there but had moved away in the past five years. We included private renters, homeowners, and people living in residences for autonomous older adults. We also conducted 10 in-depth interviews with key informants (i.e., six in La Petite-Patrie and four in Lower NDG, who came from varied backgrounds (e.g., municipal councillor, priest, community workers, etc.).  Table 1 summarizes some key characteristics of the older adults who participated in this study.

### 3.2. Data Collection

Participants were referred from a variety of community organizations (e.g., a tenant advocacy organization, the local community health care centre in both neighbourhoods, and the NDG Senior Citizens Council). Our research assistants put up posters in businesses and distributed pamphlets and presented the project at various social events for older people. We encountered important challenges recruiting individuals displaced as a result of gentrification (this is a widespread problem in gentrification research [11]). Although we had partnered with a tenant advocacy organization in La Petite-Patrie that was willing to refer recently displaced clients, this strategy only generated one interview. In Lower NDG, the Senior Citizens Council had also hoped to refer recently displaced members but was unable to locate former residents who had maintained ties with the Council. Other prospective participants were ineligible or not interested for various reasons (e.g., did not meet age requirements, insufficient length of residency, poor health). Attempts to recruit displaced individuals using the snowball method were also unfruitful. We also contacted several autonomous residences for older people in adjacent areas of Montréal, but their administrators were not willing to participate in the study. In sum, in La Petite-Patrie we interviewed five displaced residents. In Lower NDG, we interviewed one long-term resident who had recently left. However, only one of the displaced participants was forced to leave because of a housing takeover, all of the others moved due to declining health. 

Interviews were conducted in English, French, and/or Italian, lasted between 60 minutes to two hours, were voice recorded and transcribed in their entirety, the Italian material being subsequently translated into French. The original French language interview guide was developed in collaboration with our French colleagues and upon completion was translated into English and Italian. Our interviews aimed to explore what changes older residents perceive as having occurred in the neighbourhood (e.g., new constructions, population, neighbourhood image, etc.) and what the effects have been on them. Some of the potential impacts were covered systematically: social networks, change in urban landscape (e.g., loss/gain of new businesses, new constructions). We also wanted to explore to what extent older people's social networks and activities were located inside and outside of their neighbourhood, to be able to evaluate the significance of the local neighbourhood. Finally, we were interested in what place older residents see for themselves in the neighbourhood, how they feel about aging in place, what keeps them in their home. The interview ended with a brief sociodemographic questionnaire to better contextualize their perspectives. 

The key informants were interviewed using a semistructured interview guide and informed consent was obtained before each interview. The interviews lasted between 45 minutes to one hour and were voice recorded in their entirety. Participants were asked to describe the neighbourhood (types and cost of real estate and rentals, population, transportation, cultural activities, businesses, etc.). They were then asked about any changes they had noticed (new constructions, population, etc.). Finally, they were asked specifically about the role that older adults have in the neighbourhood and whether they believe the neighbourhood is a good place to age. The term “gentrification” was purposefully excluded from all recruitment material and the interview guides so as not to bias participants' responses. 

### 3.3. Analysis

All interview transcripts were read, and an analytic summary was created for each participant to understand the situation and the dynamics of social inclusion/exclusion of each of the participants. The analysis employed both deductive and inductive approaches in identifying themes to generate an understanding of how neighbourhood change was experienced in the everyday lives of older adults. The seven dimensions of the social exclusion framework [29] (symbolic, identity, territorial, sociopolitical, social network, economic, and institutional) were employed for the first round of the deductive coding. To obtain a better understanding of the participants' perspective, we also used Rowles' three components of attachment (physical, social, and autobiographical). To avoid forcing material into predefined categories and to reflect themes emerging from the data, we generated codes inductively using the grounded theory approach of Glaser and Strauss [49]. These inductively generated codes were reviewed and discussed until the research team members arrived at a consensus. The entire analysis process was facilitated by using the qualitative software package QDA Miner.

## 4. The Two Study Neighbourhoods

Before moving on to the presentation of findings, we now briefly introduce the two neighbourhoods, referring to a summary table showing how their sociodemographic characteristics evolved over the decade preceding the start of our fieldwork (Table 2).

### 4.1. La Petite-Patrie

La Petite-Patrie is a working-class inner city district a few kilometres north of downtown Montréal and dating from the 1910s–1920s when it was considered part of a larger suburban district called Villeray. It is mainly French-Canadian in ethnocultural composition but is also home to one of the founding parishes of the city's Italian-origin community. In the 1970s and 1980s its ethnocultural profile diversified with the settlement of Latin American and Southeast Asian immigrants. It is known citywide today mainly for two major culinary attractions in its western sector: the Jean-Talon produce market and the Little Italy commercial strip on nearby St. Lawrence Boulevard, both of which have been the object of large municipally led revitalization initiatives in the past 10–15 years. Little Italy has undergone a “rebranding” through ethnic entrepreneurship and many of the traditional storefronts on the main shopping street have been renovated and given way to more luxurious boutiques. Although the resident population of Italian ethnic origin has shrunk by half from 1996 (5%) to 2006 (2.5%), the local Italian business community is still powerful and Little Italy remains a draw for Italian-origin residents of Montréal and upper-middle-class consumers alike. The market has been somewhat reoriented toward regionally produced and artisanal specialty foodstuffs although a vast array of fresh produce is still available.

Bucking the societal-scale trend of an aging population, this area is now home to fewer senior citizens than a decade or so ago, and their relative weight has also diminished (Table 2). It remains ethnically diverse, although the visible minority population has fallen, especially in the sectors most touched by the commercial gentrification that has been the key change in this area over the past 15 years or so. La Petite-Patrie has seen a rapid increase in residential gentrification activity since the early 2000s, in part due to an overspill from the city's two most gentrified districts, to which it is adjacent. Housing market changes symptomatic of this gentrification are increased rates of homeownership (Table 2), spiralling real estate values (especially since the mid-2000s, according to our key informants), mushrooming infill condominium construction, and conversions of rental units and nonresidential buildings (including an iconic church) to condominiums, including some up-market housing units. Our community-based key informants claim that transformations of existing rental units have generated displacement in spite of the safeguards of tenants that are in principle built into law. As well, the neighbourhood has seen an influx of a younger, highly educated population, the average incomes of its residents, while still modest overall, have increased relative to the metropolitan area average while the proportion of low-income households has fallen (Table 2). Two community organizations that have supported our project see these changes as creating pressures on low-income renters and are especially uneasy as to whether residents in their 70s and older will still have their place in the neighbourhood if current trends persist.

### 4.2. Lower NDG

Lower NDG, an interwar suburb in the city's west end, is mainly inhabited by an English-speaking and lower- to middle-income population, but like La Petite-Patrie, it was also a major area of settlement of Italian immigrants, the wave in this case beginning in the 1940s. In this case too, the Italian-origin population has halved in the decade 1996-2006 (6% to 3.2%). Unlike La Petite-Patrie, the main socioeconomic trend over the past decade or so has been one of stability, even stagnation relative to the metropolitan area, rather than increasing income levels (Table 2). However, a mega-hospital project, the new McGill University Health Centre (MUHC) campus, was planned over a decade ago and has been under construction since 2009 on a vast site immediately adjacent to a section of this neighbourhood, named after the Catholic parish of Saint-Raymond, which forms the core of our study area. This has already led to speculative construction of condominiums, although so far these have been low end of market. Significant revitalization of this working class neighbourhood is expected once the hospital is completed. Local community organizations have been highly proactive in warning about and trying to mitigate the potentially negative effects of gentrification on the area's low-income residents. Census data for 2006 (Table 2) show a neighbourhood whose older population is holding its own in absolute terms, but not in relative terms. A major change is that the absolute and relative numbers of people belonging to a visible minority have risen considerably, unlike in La Petite-Patrie (Table 2), most of the increase being concentrated in the St-Raymond sector. As to two of the classic precursors of gentrification, the proportion of university degree holders increased faster than in the CMA as a whole but there was no relative increase in the weight of the 20–44 age group; thus, signs of incipient gentrification were less marked than in La Petite-Patrie.

## 5. Results

### 5.1. La Petite-Patrie

Before addressing the perception of changes and their assessment, it is important to describe the two main populations of older people residing in La Petite-Patrie: (1) the Italian population, homeowners who live in Little Italy, a sector within La Petite-Patrie (western sector of the neighbourhood); (2) the French-Canadians, who are mainly renters, residing near the centre and east of the neighbourhood. The Italians have a strong sense of attachment to Little Italy because this is the sector to which they immigrated, bought their first home, and raised their families (strong sense of autobiographical insideness). Several interviewees expressed their attachment to the area of Little Italy, comparing it to a village, or rather “the village”. Their lives are organized around this relatively small geographic space, and they are able to run all their daily errands on foot. Almost all of the Italians had dense social networks within the neighbourhood. They also demonstrated strong attachment to shops and cafés they frequented on a regular basis as well as the local parish and associations. The public Jean-Talon market and parks also emerged as significant places that were part of their routine. Contrary to the Italians, the French-Canadians' attachment was more instrumental: as an 85-year-old female renter pointed out, “*we appreciate the neighbourhood because everything is at your fingertips*” [translation]. Yet, some French Canadian participants expressed complex and deep-rooted attachment that went beyond the instrumental attachment to the neighbourhood. For example, the woman who was forced out of the neighbourhood because of a housing takeover continued to return frequently to shop in familiar stores. Other French Canadians felt attachment to La Petite-Patrie that went beyond having shops nearby; the attachment to their immediate environment became more evident as they expressed feeling threatened and uncomfortable with the arrival of new ethnic minorities.

For both populations, the perception of changes varied considerably among interviewees. Some participants perceived only very few changes, if any, while others perceived several. People living in social housing complexes or in older residents' apartment complexes generally perceived little change in the neighbourhood; many people living in these residences often only described the change in their individual residence. Moreover, the changes reported by participants focused on the immediate environs (one's neighbours, one's street, or at most a few surrounding streets). The most common change noticed was a perception of increasing ethnoracial diversity (which, as we saw in Table 2, is not supported by census data). Reactions to this varied from frank expressions of unease—a sense of strangeness in once-familiar public spaces which led some people not to frequent them any more—to discourses avowing tolerance and even a cosmopolitan mentality. For instance, one 79-year-old French-speaking woman stated, “*You have to go to McDonald's to see this. We don't feel at home, it's full of immigrants. I know. I don't understand how they let that many into the country! I just don't know*” [translation]. An 85-year-old French-Canadian woman said she felt “invaded,” “*It's too crowded now (St-Hubert Street), and you hear all different languages. We ask ourselves where we are. I don't like it. They are invading us! I am scared that in 10 years, what other languages are we going to hear? They are going to take everything from us … all the businesses; it's them who are running them*” [translation]. French Canadian interviewees also reported that local churches were increasingly being “taken over” by the Haitian population. Some told us they attend church much less often because they feel out of place, “*I'm the only white face in the room*” [translation], an 82-year-old woman reported. In contrast, a man of Italian descent viewed this newfound diversity positively, “*it helps to know new people, other cultures, to reduce prejudice, because we are all alike!*” [translation]. 

Several interviewees reported that real estate values and rents had increased substantially in recent years. Several also pointed out the spread of condominiums, some referring to the transformation of a local church into quite luxurious condominiums. Despite the documented increase in the number of residents with university degrees, very few study participants noted the arrival of a younger, better educated and wealthier population to the neighbourhood, with the exception of one 76-year-old French-speaking woman who welcomes the arrival of more “*refined people*” [translation]. While some owners appreciated the increased value of their homes, others saw the recent developments as negative because they did not meet the needs of families and low-income residents of the neighbourhood. An Italian-speaking owner noted that the neighbourhood has become prohibitively expensive, preventing members of his family to settle there. 

As for the business changes, the positions were also very diverse. Some participants harshly critiqued the changes to two major commercial streets in the neighbourhood, notably St-Hubert Street where the variety of its stores had been lost and the new shops did not meet the needs of the older neighbourhood residents: “*All the stores we liked, they are all gone. They were all replaced by fabric stores, prom dresses, wedding boutiques …. *“*It is not at my age that I'll buy that!*” [translation] (71-year-old French-Canadian woman, renter). As for commercial shift of St-Lawrence Boulevard and the renovation of the Jean-Talon market, many appreciated the changes and the arrival of new businesses: “*there's a lot of progress in Little Italy, in shops, restaurants … Many people are coming*” [translation] (71-year-old Italian man, homeowner). However, few say they regularly attend the new restaurants and cafés, preferring familiar places. Others lament the increased traffic in Little Italy and higher market prices that have forced them to do their shopping elsewhere. One 91-year-old Italian man (home owner) described his ambivalence about the changes: “*The Jean-Talon market used to be more traditional, now it has become very commercial, there are too many people, in the summer we can't go Fridays, Thursday evenings, and Saturday afternoon, there are just too many people! The market is working well, so for us; it is a good thing because the houses have doubled in value! To buy a home here, if I wanted to sell my house, they are going to pay!*” [translation].

A neighbourhood change that negatively affected a number of the French-Canadian residents was the disappearance of the Golden Age Clubs and bingos: “*Ah! It shocked me because it was the only fun we had. You know old people are not interested in going to bars to drink, I do not drink. That was the only place we had to go. So since it closed: “Stay home!” So we stay at home … It's as if for older residents, we're just too old, they are just waiting for us to die!*” [translation] (71-year-old French-Canadian woman, renter). In recent years, three clubs catering to French-Canadians have closed their doors, while the Italian-speaking club continues to operate. According to two key informants, these closures are related to lack of leadership in the clubs, deficient financial support from the municipal borough, and to their declining popularity, especially as the aging population of the neighbourhood decreases. In this sense, the closure may be linked to the gentrification with the arrival of a younger and more educated population.

### 5.2. Lower NDG

The two main populations residing in Lower NDG are the Italian-Canadians, of whom those in our sample are all homeowners, and the English-speaking Canadians, of whom those we interviewed are both home owners and renters (Table 1). The scarcity of businesses in Lower NDG, especially in the St-Raymond sector, forces its residents to leave the neighbourhood regularly and frequently to meet most of their consumer, social, and in some cases, spiritual needs. As such, their instrumental attachment is low, especially compared to the residents of La Petite-Patrie. Some residents expressed indifference toward their neighbourhood, as one 90-year-old English-speaking woman made clear when asked why she decided to move to the neighbourhood and stay for so many years: “*I don't know, it's a place to live, you have to live some place!*” However, some homeowners demonstrated a strong sense of attachment linked to their deep-rooted history with the neighbourhood. For example, one 74-year-old woman who bought the house she grew up in from her mother explained: “*It's just home … My family they all stuck around, you know I got 4 children. And I have the 2 boys living here. I am very … My own son with his 2 kids down the street, it's great, it's great!*” However, compared to the Italian-origin residents, the social attachments among the English speaking Canadians were more family oriented around individual homes whereas the Italians met regularly to socialize at St-Raymond's Parish and the bocce courts located behind St-Raymond's Community Centre. (“Bocce” is a ball sport belonging to the boules sport family that is commonly played outdoors during the summer months among the Italian communities in Montréal.)

As in La Petite-Patrie, the most common change noticed in Lower NDG was the increase in ethnic minority populations (which in this case is more congruent with the census data—Table 2). Perceptions were also mixed, but several participants responded negatively toward the arrival of a medical transit house for Inuit people, as they felt the centre changed the image of the neighbourhood: “*I don't know what they are going to do. I mean they (Inuit) are laying on churches … Churches lawns and … Well just yesterday, at the bus one was sitting in the door steps … I mean that's not very nice when people pass on the bus and see it, it is not very nice for people living here either …*” (74-year-old English-speaking woman, homeowner). However, some residents, such as a 90-year-old English-speaking woman renter embraced the diversity: “*Now there is everything. There's Hindus and Jewish, but everyone gets along well.*”

Some of the residents also reported feeling less secure in the neighbourhood: “*Put it this way, you ask me, if I feel at home on my street, yes. Ask me if I could go down to Saint-James, after 9 o'clock, no!*” (74-year-old English-speaking woman, homeowner). An 85-year-old English-speaking homeowner also mentioned that the neighbourhood is becoming less safe, that there have been a couple of recent shootings and a “person was beat up” at the top of her street.

Similar to La Petite-Patrie, there were also reports of lost institutions; for example, a 74-year-old English-speaking woman regrets the loss of the church in which she was an active member for over 40 years: “*I like my new church but I mean I loved my old church. That was a surprise, but I can worship anywhere. You know, it is not like I was married there, my father or mother … No they weren't married there but … But all my kids were christened there *…*, you know, so I have a lot more attachment to that, I mean to that one.*” Despite this loss, there were no collective political movements to save this important institution. Some of the English-speaking interviewees expressed frustration and felt they did not have a political voice, especially compared to the Italian population: “*It's more difficult. First of all, the older residents' voices are all Italian, who is going to give … I don't think anyone really cares. I mean politicians pretend they do for five minutes to bet whatever bills they want passed; they make it look like they really care … Yes, but this is silly, but I think there is a place for giving people more of a say in life rather than just being consumers*” (73-year-old English-speaking woman, homeowner). Despite the generally reported deterioration of the neighbourhood, the arrival of the new community centre was unanimously viewed as a positive addition, as one 73-year-old woman owner pointed out: “*Oh! It is a beautiful place.*” Participants viewed the community centre as a new place to meet with their peers, “*At least now we have a place to go in the winter, where we can go for 2-3 hours during the evening*.” [translation] (70-year-old Italian woman, homeowner). Finally, interviewees expected important future changes with the construction of the new mega-hospital centre yet the opinions were mixed. Some consider the potential negative ramifications of the construction: “*I don't know they've been talking about it for the past 10 years and nothing has happened yet. They're talking about making it (main artery running through St-Raymond) a one way street …. I can't imagine that!*” (90-year-old English speaking woman, renter). While others viewed the new construction in a more positive light: “*I think because of the super hospital I think it has given people a boost, even though there's been lots of complaints about what's being done but the fact that at least there is some activity is making the place more … less of a forgotten area … Yes it is a place that will be convenient, and ah it will be fine. Rather before it was a place that you hardly knew it existed, so now it is coming into its own.*” (73 year-old English-speaking woman, homeowner).

## 6. Discussion

Through this study we obtained an improved understanding of how older residents who are aging in place experience neighbourhoods that are themselves undergoing change. The results show that even when older residents remain in place, they may experience feelings of strangeness, insecurity, and social exclusion.

The experiences of attachment to neighbourhood differed depending on the nature of the neighbourhood and the population at hand. Instrumental (or functional) attachment was not captured by Rowles' threefold typology, yet it was the most widely reported among both the Italians and French Canadians in La Petite-Patrie. It is not surprising that there was no reported instrumental attachment in Lower NDG since the neighbourhood is lacking in local services, which forces the residents to leave the area to meet the majority of their daily needs. Commercial and institutional deficiency in Lower NDG not only prevented the residents from “living” the neighbourhood, it also prohibited them from establishing a routine with it (Rowles' [38, 39] physical insideness). However, with the arrival of the new mega-hospital, residents may become more instrumentally attached, as some of the residents forecast that the construction will bring new businesses and services to their “forgotten” neighbourhood.

Two groups seem to stand out in both neighbourhoods, although we are cautious given the small number of participants: (1) the Italian homeowners, and (2) the English and French-speaking renters. First, neighbourhood attachment is recognized as being more prevalent for homeowners than it is for renters, the formers' symbolic as well as material investment being greater due to their likelihood of moving less frequently than renters [45]. Second, compared to the Italians, the French- and English-speaking Canadian interviewees had a widely dispersed social network in the city, and/or had moved several times during their adult lives, thereby not establishing the same sense of social connectedness (social insideness) as the Italians. The Italians' description of the neighbourhood as a “village” effectively portrays their warm feelings of connectedness with neighbours and their surroundings and seems to be related to their stronger sense of autobiographical insideness, as they had built their lives in the neighbourhood. In Lower NDG, some of the English-speaking Canadians were homeowners, yet there was much less a sense of social connectedness and autobiographical insideness beyond their individual homes and their immediate families.

We have alluded to the fact that the older residents' perceived changes do not necessarily reflect the reality of the neighbourhood changes, especially in regards to the proportion of the visible minority population (principally in La Petite-Patrie). Yet the first, and sometimes the only, type of change noted in both neighbourhoods was in terms of visible signs of an increasing ethnoracial diversity and physical signs such as new condos, commercial revitalisation, and so forth. On the other hand, new younger and better-educated populations went virtually unnoticed. This relative invisibility of the arrival of this new population suggests that social class change is less dramatic than ethnic distinctions. This finding draws a parallel to Alba's [50] Mexican study, where changes related to gentrification were not perceived by interviewees besides home renovations. Negative comments relating to the perceived increase in ethnic minorities were more common than were positive ones. The French Canadians in La Petite-Patrie and the English-speaking participants in Lower NDG felt especially “invaded,” that they no longer belonged or felt “at home” among the new faces on their once familiar landscape. The negative feelings of “strangeness” in a well-known environment provide evidence of indirect displacement and symbolic exclusion [29] resonating with Nord's [51] “politics of resentment” in which the London shopkeepers who were interviewed blamed multiculturalism and cultural diversity in the neighbourhood for inequalities and feeling powerless. On the other hand, the Italians did not report the same negative experience, which is likely to be related to their strong sense of social insideness to the neighbourhood.

Among the reported changes, the greatest effect on older residents was related to the closure of the Golden Age Clubs and churches as well as the revamping of a commercial street (niche market of bridal and evening wear) and a public market (higher market prices). However, an important distinction between the English- and French- speaking populations was that the English-speaking residents of Lower NDG seemed less affected by changes, such as the closure of their Anglican church, because they were used to leaving the neighbourhood to meet their needs. In addition, an unexpected result was the implementation of a new community centre with activities catering to older people in Lower NDG, a deteriorating neighbourhood, whereas in La Petite-Patrie, a neighbourhood undergoing gentrification, we see the closure of important institutions for older adults. This surprising finding is at odds with the viewpoint of Bowling and Stratford (2007) [52] who suggest that increasing the affluence of an area may improve the social and physical functioning of older people who are aging in place. This was not the case in La Petite-Patrie, where the closure of French-Canadian Golden Age Clubs led to a form of “house arrest” for some of the participants. Yet, in Lower NDG, the English-speaking Canadians reported fear of crime, which prevented them from going out at night. As noted by Anne-Marie Séguin et al. [53], when one is confined to the home it becomes a place of isolation and invisibility. Feelings of insecurity and the disappearance of familiar institutions provided evidence of Billette and Lavoie's [29] dimension of territorial exclusion. Conversely, in both neighbourhoods, the Italians had managed to maintain their social and cultural institutions. For instance, at present, the Italian Parish in La Petite-Patrie has many members who attend very regularly, and a number of activities continue to be organized around the church. Similarly, the Italian Golden Age clubs of this community continue to operate, contrary to the French-Canadian clubs. A key informant even spoke of the older Italians wanting to open a residence catering specifically to Italian seniors in Little Italy. Similarly, in Lower NDG, the older Italians continued to meet regularly at St-Raymond's Church and the bocce courts located behind St-Raymond's Community Centre. 

For a number of reasons, the residents of Italian descent in both neighbourhoods viewed the neighbourhood changes with more serenity and comfort than the French- and English-speaking Canadian residents. Similar to Pashup-Graham [28] whose Chicago-based study unveiled some of the positive consequences of gentrification, the Italians viewed the revitalization with enthusiasm; they recognized that the neighbourhood was becoming more attractive and that the value of their homes was increasing. While the Italians were mostly homeowners, giving them some protection against the gentrification of the neighbourhood, the French Canadians were all renters. It is thus not surprising that the renters we met, be they French or English-speaking, were at the same time less attached to their neighbourhood and possibly more vulnerable to perceived and objective local changes generating experiences of social exclusion. For instance, the French- and English- speaking Canadians were experiencing forms of symbolic exclusion, as was pointed out by two key informants who believed that these populations were no longer seen or heard, rendering them invisible. Similar to Martin [23] who looks at political displacement, the absence of the voices of this population in politics and decision making also suggests a form of sociopolitical exclusion. The visibility and political influence of the Italians were obvious to some of the other interviewees, which may have reinforced feelings of exclusion among the French- and English-speaking populations. This finding is supported by Phillipson [14] who writes, “variations in community attachments now illustrate significant inequalities within the older population: most notably between those able to make conscious decisions about where and with whom to live, and those who feel marginalised and alienated by changes in the communities in which they have “aged in place” (page 336). Finally, unlike the French- and English-speaking communities, the Italians had managed to maintain their cultural and social institutions. The reasons for this preservation are complex, and we are cautious given the small sample size; it appears that their strong sense of physical, social, and autobiographical insideness led to greater visibility, political power, and control over changes, which in turn protected them from some dynamics of social exclusion.

The first expected impact of gentrification is often financial [54]. An unexpected finding was that almost no respondents experienced economic exclusion. This appears to be related to the fact that La Petite-Patrie is undergoing incomplete gentrification [55]; that the neighbourhood is maintaining a certain social mix that is manifested by the heterogeneity of businesses, the cost of housing, and social status of the population. A second potentially protective element is that Québec has a system of rent regulation. The situation could be very different in other cities that do not have these protective measures in place; thus there is scope for further research. 

## 7. Conclusion

The majority of environmental gerontology research has focused on how to provide security and strengthen an older person's sense of self while they age in place. There is a call for further research that considers how neighbourhood change affects older residents who age in place. This study goes beyond economic impacts of neighbourhood change and considers the importance of social, cultural, and political consequences that may affect people's quality of life. Our observations also support the relevance of examining the possible role of gentrification in the dynamics of social exclusion of older people who are living in a changing working class neighbourhood and, at the same time, have little control over local institutions and organizations that are essential to meet their needs. In addition, this study reinforces the importance of considering the heterogeneity of the older adult population; inequalities and social differences still exist, even within golden age cohorts. To this end, Manzo [56], citing Hummon [56], recalled that the rootedness of some members of the community involves the removal and exclusion of other members. Finally, our findings demonstrate the crucial role that social spaces play in order to maintain or develop social links, increase visibility and consequently feelings of inclusion. There is a need to maintain these social spaces for older residents, especially in changing environments, to ensure that older people have a space to be seen and heard.

## Figures and Tables

**Table 1:** Profile of study participants (*n* = 30).

	La Petite-Patrie	Lower NDG	Total
*Total participants*	18	12	30
Men	6	5	11
Women	12	7	19

65–69	1	0	1
70–74	1	3	4
75–79	8	3	11
80–84	4	1	5
85–90	3	3	6
90+	1	2	3

*Mother tongue*			
French	12	0	12
English	0	5	5
Italian	6	7	13

Highest level of education*			
Primary school (incomplete or complete)	8	7	15
Some high school	5	1	6
High school (completed)	2	1	3
Postsecondary	2	2	4

*Socioeconomic status*			
Low income^#^	9	3	12

*Current residents*			
Owners	5	9	14
Renters	13	3	16
Renters living in HLM (public housing)^∞^	4	1	5

*Former residents*	5	1	6
*Years in neighbourhood * ^*£*^			
Less than 30 years	5	4	9
30 to 39 years	7	2	9
50 years and over	6	6	12
Total	18	12	30

*No information for two participants, one in each neighbourhood.

^#^For the study purposes, low-income participants are those receiving the guaranteed income supplement (GIS), which provides additional money to top off the Old Age Security Pension. The maximum annual income for a single person GIS recipient is $15,960 (Service Canada, Old Age Security Payment Rates, April-June 2011: http://www.servicecanada.gc.ca/eng/isp/oas/oasrates.shtml). This definition is more stringent than Statistics Canada's low-income cut-off of $22,229 before tax in 2009 for a single person living in a city of more than 100,000 inhabitants (Statistics Canada, Low Income Lines 2008-2009: http://www.statcan.gc.ca/pub/75f0002m/2010005/tbl/tbl02-eng.htm).

^∞^HLM (Habitations à loyer modique) are apartment complexes for low-to-modest income households, owned and managed by the public sector. Rent is set at 25% of household income and includes basic utilities. Tenants are selected from a waiting list according to needs-based criteria established by the provincial government. Those in our study are specifically for autonomous older adults.

^*£*^Lowest value of years in neighbourhood is 9 years followed by 12 years; all others resided in neighbourhood over 15 years.

**Table 2:** Basic Sociodemographic data, Montréal Census Metropolitan Area (CMA), Lower NDG, and La Petite-Patrie, 1996 and 2006.

	Montréal CMA		Lower NDG		Petite-Patrie	
	1996	2006	1996	2006	1996	2006
Total population	3,326,510	3,635,571	9,553	10,284	15,792	15,423
Variation %		+9.3		+7.7		−2.3
Population 65 and over	400,135	495,690	1,110	1,120	2,025	1,740
% of total population	12.2	13.6	11.6	10.9	12.8	11.3
Population 20 to 44	1,338,110	1,313,615	4680	4995	7735	8225
% of total population	40,2	36,1	49,0	48,6	49,0	53,4
% with university degree	15.4	21.0	21.1	29.8	15.4	31.3
% low income households	27.3	21.1	45.6	41.8	58.6	40.4
Average total personal income ratio (CMA = 1.0)	1.0	1.0	0.75	0.74	0.60	0.72
% of private dwellings owned	48.5	53.4	19.0	20.3	15.5	18.5
Visible minority population	401,420	590,375	2,695	3,605	4,150	3,730
% of total population	12.2	16.5	28.7	35.8	26.5	24.4

Source: Statistics Canada, Censuses of 1996 and 2006, 20% sample data. The data for the case study neighbourhoods were calculated by aggregation of data published at the census tract level of geography.
